# PecanPy: a fast, efficient and parallelized Python implementation of *node2vec*

**DOI:** 10.1093/bioinformatics/btab202

**Published:** 2021-03-24

**Authors:** Renming Liu, Arjun Krishnan

**Affiliations:** Department of Computational Mathematics, Science and Engineering, Michigan State University, East Lansing, MI 48824, USA; Department of Computational Mathematics, Science and Engineering, Michigan State University, East Lansing, MI 48824, USA; Department of Biochemistry and Molecular Biology, Michigan State University, East Lansing, MI 48824, USA

## Abstract

**Summary:**

Learning low-dimensional representations (embeddings) of nodes in large graphs is key to applying machine learning on massive biological networks. *Node2vec* is the most widely used method for node embedding. However, its original Python and C++ implementations scale poorly with network density, failing for dense biological networks with hundreds of millions of edges. We have developed PecanPy, a new Python implementation of *node2vec* that uses cache-optimized compact graph data structures and precomputing/parallelization to result in fast, high-quality node embeddings for biological networks of all sizes and densities.

**Availabilityand implementation:**

PecanPy software is freely available at https://github.com/krishnanlab/PecanPy.

**Supplementary information:**

Supplementary data are available at *Bioinformatics* online.

## 1 Introduction

Large-scale molecular networks are powerful models that capture interactions between biomolecules (genes, proteins, metabolites) on a genome scale (McGillivray *et al*., 2018) and provide a basis for predicting novel associations between individual genes/proteins and various cellular functions, phenotypic traits and complex diseases (Liu *et al*., 2020; Sharan *et al*., 2007). An area of research that has gained rapid adoption in network science across disciplines is learning low-dimensional numerical representations, or ‘embeddings’, of nodes in a network for easily leveraging machine-learning (ML) algorithms to analyze large networks (Cai *et al.*, 2018; Goyal and Ferrara, 2018; Hamilton *et al*., 2018). Since each node’s embedding vector concisely captures its network connectivity, node embeddings can be conveniently used as feature vectors in any ML algorithm to learn/predict node properties or links (Hamilton *et al.*, 2018). One of the earlier node embedding methods that continues to show good performance in various node classification tasks, especially on biological networks (Liu *et al.*, 2020; Nelson et al., 2019; Yue *et al*., 2019), is a random-walk based approach called *node2vec* (Grover and Leskovec, 2016). Recent studies on the task of network-based gene classification have shown that *node2vec* achieves the best performance among the state-of-the-art embedding methods for gene classification (Yue *et al.*, 2019), and that using embedding generated from *node2vec* achieves prediction performance comparable to the state-of-the-art label propagation methods (Liu *et al.*, 2020).

However, despite its popularity, the original *node2vec* software implementations [written in Python (https://github.com/aditya-grover/node2vec), and in C++ (https://github.com/snap-stanford/snap/tree/master/examples/node2vec)] have significant bottlenecks in seamlessly using *node2vec* on all current biological networks. First, due to inefficient memory usage and data structure, they do not scale to large and dense networks produced by integrating several data sources on a genome-scale (17–26k nodes and 3–300mi edges) (Greene *et al*., 2015; Szklarczyk *et al*., 2015). Next, the pleasingly parallel precomputations of calculating transition probabilities and generating random walks are not parallelized in the original software. Resolving these issues will make it possible to embed large and dense biological networks, and even large biological knowledge graphs. Finally, the original implementations only support integer-type node identifiers (IDs), making it inconvenient to work with molecular networks typically available in databases where nodes may have non-integer IDs.

Recent work presented in preprints (Zhou *et al*., 2018) and unpublished code repositories have proposed other implementations of *node2vec* (Supplementary Table S1). However, they either do not provide publicly available software or do not present a full analysis of their implementation, including a benchmark that ensures the quality of the resulting embeddings. Here, we present PecanPy, an efficient Python implementation of *node2vec* that is parallelized, memory efficient and accelerated using Numba with a cache-optimized data structure (Supplementary Fig. S1). We have extensively benchmarked our software using networks from the original study and multiple additional large biological networks to demonstrate both the computational performance and the quality of the node embeddings. In the rest of this paper, we first summarize the optimization and the performance of PecanPy and then go into the details of our implementation.

## 2 Implementation notes

The *node2vec* program consists of four stages: loading, preprocessing, walking and training (detailed description of the *node2vec* software is in Supplementary Notes). Comprehensive evaluation of the four stages (Supplementary Figs S2–S6) shows that training only takes 1.2% (median) of the total runtime for the original Python implementation, in contrast to 95.1% for the original C++ implementation. The raw training runtimes indicate that training the skip-gram using the gensim Python package is consistently an order of magnitude faster than the original C++ implementation. We chose to focus on Python because it is currently the most widely used high-level language in machine learning, making it convenient to use and to develop further as part of the community. Further, the Numba compiler can be used to achieve C++ level performance. We reimplemented *node2vec* in Python and optimized the first three inefficient stages of the algorithm by: (i) implementing computationally and memory-optimized graph data structures with efficient loading strategies; (ii) providing an option to bypass the need to store all transition probabilities, leading to a significant reduction in memory usage; and (iii) parallelizing the processes of transition probability computation and walk generation. We have also made string-type node IDs acceptable.

We present these improvements as PecanPy, a new software for **p**arallelized, **e**ffi**c**ient and **a**ccelerated ***n****ode2vec* in **Py**thon (Supplementary Fig. S1). PecanPy operates in three different modes—*PreComp*, *SparseOTF* and *DenseOTF—*each optimized for networks of different sizes and densities (Supplementary Table S2). *PreComp* precomputes and stores all second order transition probabilities as in the original implementations, and hence is more suitable for small and sparse networks. On the other hand, *SparseOTF* and *DenseOTF* both compute second order transition probabilities On-The-Fly during walk generation without saving them. *SparseOTF* (like *PreComp*), with its use of a compact sparse row matrix representation, is better-suited for networks that are large and sparse. *DenseOTF*, which uses the full adjacency matrix as the underlying graph data structure, is well-suited for dense networks. The different modes and optimizations are detailed in the Supplementary Notes.

## 3 Benchmarks

**Fig. 1.**
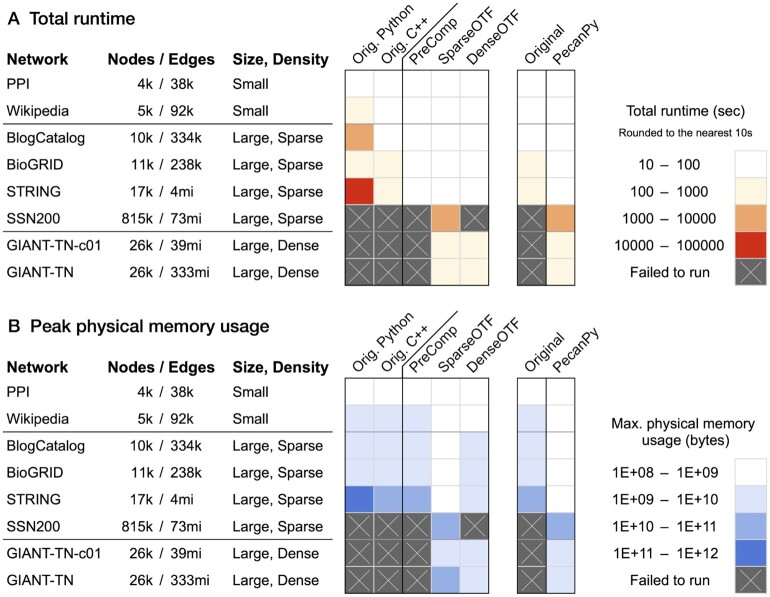
Summary of runtime and memory of PecanPy and the original implementations of node2vec using multiple cores. The eight networks of varying sizes and densities are along the rows. The software implementations are along the columns. The first heatmap (on the left) shows the performance of the original Python and C++ software along with the three modes of PecanPy (PreComp, SparseOTF and DenseOTF). The adjacent 2-column heatmap (on the right) summarizes the performance of the original (best of Python and C++ versions) and PecanPy (best of PreComp, SparseOTF and DenseOTF) implementations. Lighter colors correspond to lower runtime in (**A**) and lower peak physical memory usage in (**B**). Crossed gray indicates that the particular implementation (column) failed to run for a particular network (row)

We comprehensively benchmarked PecanPy and the original Python and C++ implementations of *node2vec* on a collection of eight networks including three networks from the original *node2vec* paper (Grover and Leskovec, 2016) and five large biological networks that together span a wide range of sizes (∼4k to 800k nodes and ∼38k—333mi edges) and densities (0.02–100%; Supplementary Table S3; Fig. 1, Supplementary Figs S7–S9; see Supplementary Notes on the choice of implementations) (Greene *et al.*, 2015; Law *et al*., 2020; Stark *et al*., 2006; Szklarczyk *et al.*, 2015). All software testing was done using 28 core Intel Xeon CPU E5-2680 v4 @2.4 GHz.

First, across the board, PecanPy (in one of its three modes) is substantially faster than the original implementations. In fact, there are three large networks (SSN200, GIANT-TN-c01, GIANT-TN) that run successfully only using PecanPy’s *OTF* implementations. Other implementations failed to run the GIANT-TN network due to memory limitations that arise from storing second-order transition probabilities. The original software failed to run SSN200 because they do not support non-integer-type node IDs; this is supported in PecanPy. *DenseOTF* failed for SSN200 since its dense-network design requires more than 5TB of memory to create a double precision dense matrix of size 800k. However, it considerably improves memory usage and speed for large dense networks like GIANT-TN, due to the more efficient network loading scheme, i.e. reading a numpy array file instead of a text (edge list) file (Supplementary Fig. S4). For relatively small and sparse networks (e.g. BioGRID, BlogCatalog), using PreComp invariably results in faster walk generation, thus achieving an overall shorter runtime (Supplementary Fig. S4). These results underscore the importance of the three modes of PecanPy. Similarly, in terms of memory usage, one of the modes of the PecanPy reduces the maximum resident size by up to two orders of magnitude compared to the original implementations. All these trends are magnified on a single core (Supplementary Figs S5, S7 and S9). Another implementation nodevectors that handled at least one dense network better than the original implementations still overall performed worse than PecanPy (Supplementary Fig. S10).

Finally, to ensure the quality of node embeddings generated by our new implementations, we evaluated their use as feature vectors in node classification tasks using datasets from the original paper (BlogCatalog, PPI, Wikipedia; see *Methods*). As shown in Supplementary Figure S11, our implementations achieve the same performance as the original Python implementation (complete Wilcoxon statistics and description can be found in Supplementary Table S5 and Supplementary Notes). On the other hand, the original C++ implementation is significantly worse than the original Python implementation for PPI (Wilcoxon *P*-value = 1.98e-3), while being significantly better for Wikipedia (Wilcoxon *P*-value = 2.41e-4). These differences are likely due to the different skip-gram implementations in the C++ and the Python implementations. The performance of nodevectors feature vectors is worse than those from all other implementations. The code for reproducing all the benchmarking results presented here are available at https://github.com/krishnanlab/PecanPy_benchmarks.

## 4 Conclusion

We have developed an efficient *node2vec* Python software—PecanPy—with significant improvement in both memory utilization and speed. Extensive benchmarking demonstrates that PecanPy efficiently generates quality node embeddings for networks at multiple scales including large (>800k nodes) and dense (fully connected network of 26k nodes) networks that the original implementations failed to execute. PecanPy is freely available at https://github.com/krishnanlab/PecanPy, can be easily installed via the pip package-management system (https://pypi.org/project/pecanpy/), and has been confirmed to work on a variety of networks, weighted or unweighted, with a wide range of sizes and densities. Therefore, it can find broad utility beyond biology.

## Supplementary Material

btab202_Supplementary_Data
