# Supplementary material for: PecanPy: a fast, efficient and parallelized Python implementation of node2vec
Source: Bioinformatics. 2021 Mar 24;37(19):3377–9. doi: 10.1093/bioinformatics/btab202 (PMC8504639; doi:10.1093/bioinformatics/btab202)
Supplement: btab202_Supplementary_Data [file btab202_supplementary_data.zip › PecanPy_Supplement_R1.pdf]

1. Supplementary Notes

A Brief Description of *node2vec* and its Utility in Biology and Network Science

The *node2vec* algorithm (Grover and Leskovec, 2016) is the most widely used method to create numerical distributed representations of nodes in a network (graph) called node embeddings. *Node2vec* creates node embeddings based on generating a corpus of random walks on the input graph (Hamilton *et al.*, 2018). In contrast to earlier approaches such as *DeepWalk* (Perozzi *et al.*, 2014) that perform 1st order random walk, *node2vec* performs 2nd order random walk where the transition probability from a current node to the next node also depends on the previous node that was visited. This 2nd order random walk strategy allows the flexibility of choosing between search strategies such as Breadth First Search and Depth First Search by setting the values of the return parameter and in-out parameter. These 2nd-order walks are then fed to the skip-gram model in the *word2vec* algorithm (Mikolov *et al.*, 2013) to compute the vector representation of each node.

Numerical representations of nodes created by methods like *node2vec* open-up the possibility of deploying any tool in modern statistical or machine learning toolkit to analyze node or network properties beyond the traditional techniques of graph theory. Hence, node embedding, particularly using *node2vec*, is gaining rapid adoption in the analysis of biological networks (Nelson *et al.*, 2019), especially for the task of node classification (Yue *et al.*, 2020). Some examples of this general task are: 1) classifying uncharacterized genes in a functional interaction network to cellular functions they might participate in (Liu *et al.*, 2020), and 2) classifying medical terms in a term co-occurrence network (mined from electronic health records) to semantic types (e.g. ‘drug’, ‘disease’, ‘symptoms’, etc.) (Finlayson *et al.*, 2014).

Further, networks/graphs representations are used in nearly every discipline including sociology, economics, and language (Leskovec and Krevl, 2014). These fields are also rapidly adopting embedding-based approaches. In all cases, with better technology and dropping costs, the ability to generate and collate massive amounts of raw data is increasing rapidly, which in turn results in ever larger networks (hundreds of thousands of nodes) with greater densities (hundreds of millions of edges) across application areas. Therefore, continuing to apply techniques such as node embedding requires software that scales reasonably with these growing large, dense networks.

Here, we present PecanPy, a general software that serves this exact critical need (**Fig. S1**). It is the fastest implementation of *node2vec* that achieves its speed and efficiency by overcoming all the bottlenecks in the original implementations (**Fig. 1, S2, S3**, **S4**, **S5**, and **S6**). The details of PecanPy’s implementation – including parallelization, memory efficiency, and acceleration using Numba with a cache-optimized data structure (**Table S2**) – are given below.

Choice of *node2vec* software implementations

We first compiled a list of existing *node2vec* implementations (**Table S1**) and noted that beyond the original implementations (in Python https://github.com/aditya-grover/node2vec and C++

https://github.com/snap-stanford/snap/tree/master/examples/node2vec), none of the other implementations have attributes that are likely to improve their speed and memory usage over and above the original Python or C++ implementations with the exception of one implementation called nodevectors (https://github.com/VHRanger/nodevectors). Benchmarking for overall speed and memory showed that nodevectors handled at least one dense network better than the original implementations (**Fig. S10**). However, node embedding vectors from nodevectors resulted in very poor performance of node classification models (**Fig. S11**). Therefore, we decided to conduct all the detailed, stage-by-stage benchmarking of PecanPy only against the original Python and C++ implementations.

Networks

We used a collection of eight networks for testing and benchmarking all *node2vec* implementations. **Table S3** contains summary statistics of these networks including number of nodes, edges, and network density. PPI, BlogCatalog, and Wikipedia are from the original *node2vec* paper (Grover and Leskovec, 2016) (download link from *node2vec* webpage https://snap.stanford.edu/node2vec/). BioGRID (Stark *et al.*, 2006), STRING (Szklarczyk *et al.*, 2015), and GIANT-TN (Greene *et al.*, 2015) are molecular interaction networks (download from https://doi.org/10.5281/zenodo.3352323), where nodes are proteins/genes and edges are interactions between them. GIANT-TN-c01 is a sub-network of GIANT-TN where edges with edge weight below 0.01 are discarded. SSN200 (Law *et al.*, 2019) is a cross-species network of proteins from 200 species (download from https://bioinformatics.cs.vt.edu/~jeffl/supplements/2019-fastsinksource/), with the edges representing protein sequence similarities.

Benchmarking Runtime and Memory Usage

We profiled the runtime and memory usage for each implementation on each network. All tests were performed using a 28 core Intel Xeon CPU E5-2680 v4 @2.4GHz. Each one of four stages in the *node2vec* software was timed individually. To profile the original implementations, we added timers to each stage using the built-in time function in Python and C++. Memory was profiled using the system built-in GNU timer, which could measure the maximum resident size (physical memory usage) throughout the runtime of a program. Runtime and memory profiling were carried out with two different resource configurations. The results in the main paper are based on a multi-core configuration, with 28 cores, 200GB allocated memory, and a 24-hour time limit. We also profiled the implementations in a single-core configuration, with 1 core, 32 GB memory allocated, and 8 hours time limit. The multi-core setup emulates performance on a high-performance computing facility, while the single-core setup emulates the scenario of the software being run on a personal computer. All testing results can be found in **Table S4**.

Efficient Graph Data Structure to Improve Loading Networks

The first improvement we made was to implement a more efficient graph data structure for loading networks into the software. In the original Python implementation, *NetworkX*, which implicitly assumes that the input is a multigraph, was used to handle all operations on networks

using a graph object in the form of nested dictionaries (dict-of-dict-of-dict; (Hagberg *et al.*, 2008)). The levels of these dictionaries correspond to node, neighbors of node, edge type, and edge weight. Thus, explicit declaration of edge types for weighted edges is required. However, *node2vec* only deals with homogeneous networks where only one type of edge is present in the network. In the original Python implementation, all edge types are set to "weight" by default. This extra piece of information requires 295 additional bytes of memory for every single edge stored in a *NetworkX* graph (empty dictionary = 240bytes, empty string = 49bytes, single character = 1byte). This requirement not only causes memory overheads, but also computation overheads by reading the dictionary for "edge type" that is irrelevant for homogeneous networks. To address these issues, in this work, we implemented a lite graph object as a network loader in the form of list-of-dict, which assumes the network has only one type of edge. As shown in **Figure S6** (first and second bars in each group), both the loading time and memory usage for list-of-dict were significantly reduced compared to that for *NetworkX*. Thus, the lite graph object efficiently loads networks with reduced memory usage and shorter load time compared to *NetworkX*.

Cache Optimization to Further Optimize Graph Data Structure

Next, we optimized the graph data structure further for better cache utilization during computation. Despite being able to load faster with less memory usage using list-of-dict, operating on Python dictionaries is still suboptimal from the perspective of cache utilization. Specifically, the neighboring-edge data, which are often used together to compute transition probabilities, are not physically close to each other in memory. The design principle behind cache, however, is that units of memory that are physically nearby are likely to be used together. Consequently, every time a specific piece of data in memory is accessed, a chunk of physical memory that sits right next to the desired memory – called the cache line – is also copied from RAM to cache. Moreover, reading from cache could be up to 100 times faster than reading from RAM. To fully leverage the spatial locality of cache lines, inspired by a recent blog post (https://www.singlelunch.com/2019/08/01/700x-faster-node2vec-models-fastest-random-walks-on-a-graph/), we further converted the list-of-dict graph data structure to the compact sparse row (CSR) format, implemented using NumPy arrays (Walt *et al.*, 2011). In this way, neighboring-edge data is placed physically close together, thus improving cache utilization. *PreComp* uses CSR as their underlying graph data structure. These optimizations led to speedups in the preprocessing (**Fig. S5C**) and walk generation (**Fig. S5B**) steps for *PreComp* compared to that for the original Python implementation in the single-core setup. This includes up to an order-of-magnitude speedup for preprocessing by *PreComp* on both the BlogCatalog and Wikipedia networks.

Due to the compactness of the CSR representation of sparse matrices, memory usage can be further reduced compared to list-of-dict, as shown in **Figure S6B** (this bar in each group). However, for the same reason of compactness, dynamically constructing sparse matrices using CSR is extremely inefficient and expensive. Hence, we first use list-of-dict to load the network

into memory and then convert the full network to CSR, on which the computation will be performed.

Optimization for Dense Networks

The CSR representation described above is memory-efficient only if the network is relatively sparse because it stores non-zero entries of the adjacency matrix using both the indices and the weights of edges. For dense networks, explicit indexing of edges would cause memory overhead for the same reasons that redundancy of edge-type information (discussed before) would strain memory. To address this issue, for dense networks like GIANT-TN (25,825 nodes, fully connected and weighted; 333,452,400 edges), we implemented a dense NumPy matrix instead of CSR as the graph data structure.

CSR and dense matrix result in roughly similar walking times, as can be seen from comparing *SparseOTF* (that uses CSR) to *DenseOTF* (**Fig. S2B**). The benefit of using the dense matrix data structure over CSR instead comes from faster network loading and less memory usage. For dense networks, loading time takes up most of the runtime. For example, in the multi-core setup, loading the GIANT-TN network as CSR contributes to nearly 80% of the runtime (**Fig. S2H**). This burden is mostly due to the inefficiency in reading edgelist files as text line-by-line. To mitigate this issue of long loading time for dense networks, we implemented an option in our software that offers users the ability to first convert the edgelist file to dense matrix format and save as a binary Numpy npz file, which could then be loaded as a network in the future. As shown in **Figure S6A**, for sparse networks like PPI, loading networks as CSR is faster than loading as npz files, but as the density of network increases, loading networks as npz files becomes a better option. Similar arguments could be made for memory usage (**Fig. S6B**). For GIANT-TN (**Fig. S4D** *SparseOTF* vs *DenseOTF*), loading as npz only took 9 seconds with 6GB of peak memory usage, resulting in a 70x speedup over CSR, which took more than 10 mins to load with 39GB of peak memory usage.

Then, the question is, when exactly is using a dense matrix more optimal than using CSR. A dense matrix requires $8 \times N^2$ bytes of memory, while a CSR requires roughly $12 \times E$ bytes of memory (using 32 bit unsigned integer as index and 64 bit floating point number as data), where N is the number nodes and E is the number of edges. Hence, in theory, for any network with density less than two-thirds, CSR should be used over the corresponding dense matrix. However, since CSR cannot be directly loaded but requires an intermediate conversion using list-of-dict, the peak memory usage is also affected by the list-of-dict graph data structure. Based on the observations from **Figure S6B**, where the fold difference in peak memory usage between CSR and Numpy matrix is much smaller for other sparse networks like BioGRID, we empirically set the balancing point for network density to be around 1/10.

Memory Usage

Next, we focussed on reducing memory usage by computing 2nd order transition probabilities on the fly. The original *node2vec* implementations precompute and store all the 2nd order transition probabilities in advance, which takes up at least $\frac{E^2}{N}$ space in memory. One remedy for this issue

with high memory usage is to calculate the 2nd order transition probabilities On-The-Fly (*OTF*) during walk generation without saving them. Another reason for not precomputing 2nd order transition probabilities is that, as the network becomes larger and denser, it is very likely that most of the pre-calculated 2nd order transition probabilities are never used in the generation of walks, causing not only the space, but also the invested computation time to be wasted. We combined the CSR and the dense matrix representation each with the *OTF* strategy into separate modes in our software called *SparseOTF* and *DenseOTF*, respectively. As an instance of improvement of the OTF strategy, the *DenseOTF* implementation was able to embed a dense network like the ~26k fully-connected weighted GIANT-TN network (>333 million edges) in just an hour with only 6GB of peak memory usage, using a single core (**Table S4**). Remarkably, this means that even for an extremely dense and large network like GIANT-TN, the software could be run on a personal computer configured with a reasonable amount of memory (e.g. 16GB). Either of the original *node2vec* implementations failed to run even the sparsified version GIANT-TN-c01 (similar number of nodes and ~11% of the edges as GIANT-TN) on a supercomputer configured with 200GB memory. Moreover, the runtime of 1 hour for embedding the GIANT-TN network using *DenseOTF* with single-core configuration is even shorter than that for embedding a significantly smaller and sparser network STRING (67% of the nodes and 1% of edges as in GIANT-TN) using the original Python implementation with 28 cores, which took 5 hours to finish (**Table S4**).

For relatively small and sparse graphs where the amount of memory on a computer is sufficient to fit all 2nd-order transition probabilities, it could indeed save time generating walks by avoiding redundant computations for transition probabilities (**Fig. S2B**). Hence, we leave the decision for the trade off between speed and memory to the user by having both precomputation (*PreComp*) and the on-the-fly (*SparseOTF, DenseOTF*) schemes as options in the software.

Parallelism

Finally, all the modes of our new *node2vec* implementations are fully parallelized. As mentioned earlier, the process of computing transition probabilities and the process of generating walks for each node are embarrassingly parallel. Specifically, in the process of walk generation, each node is used as a starting point for an independent random walk with fixed length (unless a dead end is reached, causing early stopping) on the network. This process is repeated multiple times depending on the input parameter specified by the user. As each of these random walks are independent, multiple walks can be performed in parallel. Similarly, the precomputation of 2nd-order transition probabilities is only dependent on the 1st-order transition probabilities. Hence, in this work, the process of walk generation and 2nd-order transition probabilities precomputation are parallelized using Numba.

Evaluating the Quality of Node Embeddings

The three networks used in the *node2vec* paper (PPI, BlogCatalog, Wikipedia) come with labels associated with each node. Performance of node classification tasks using the generated embeddings from different implementations are compared using the three networks. Some of the labelsets from the data repository appeared to have very few positive examples (e.g. 1). For

more rigorous evaluation, only labelsets with at least 10 positive examples are evaluated. There are 38 node classes in BlogCatalog, 50 node classes in PPI, and 21 node classes in Wikipedia. For each labelset (node class), a one vs rest L2 regularized logistic regression model is trained and evaluated through 5-fold cross validation. Each test fold is evaluated by auROC separately, and the mean auROC score across the 5 folds is reported. This process is repeated 10 times for each labelset and the mean value of the reported scores are taken as the final evaluation score, which corresponds to a single point the **Fig. S11**. Using the above evaluation procedure, for each network, each implementation has a list of auROC scores depending on the number of node classes in the network. For each prediction task, we used a Wilcoxon paired test to compare this list of scores to that of the original Python implementation. The resulting statistics are used as a measure of embeddings generated from a given implementation. The full wilcoxon statistics can be found in **Table S5**.

2. Supplementary Figures

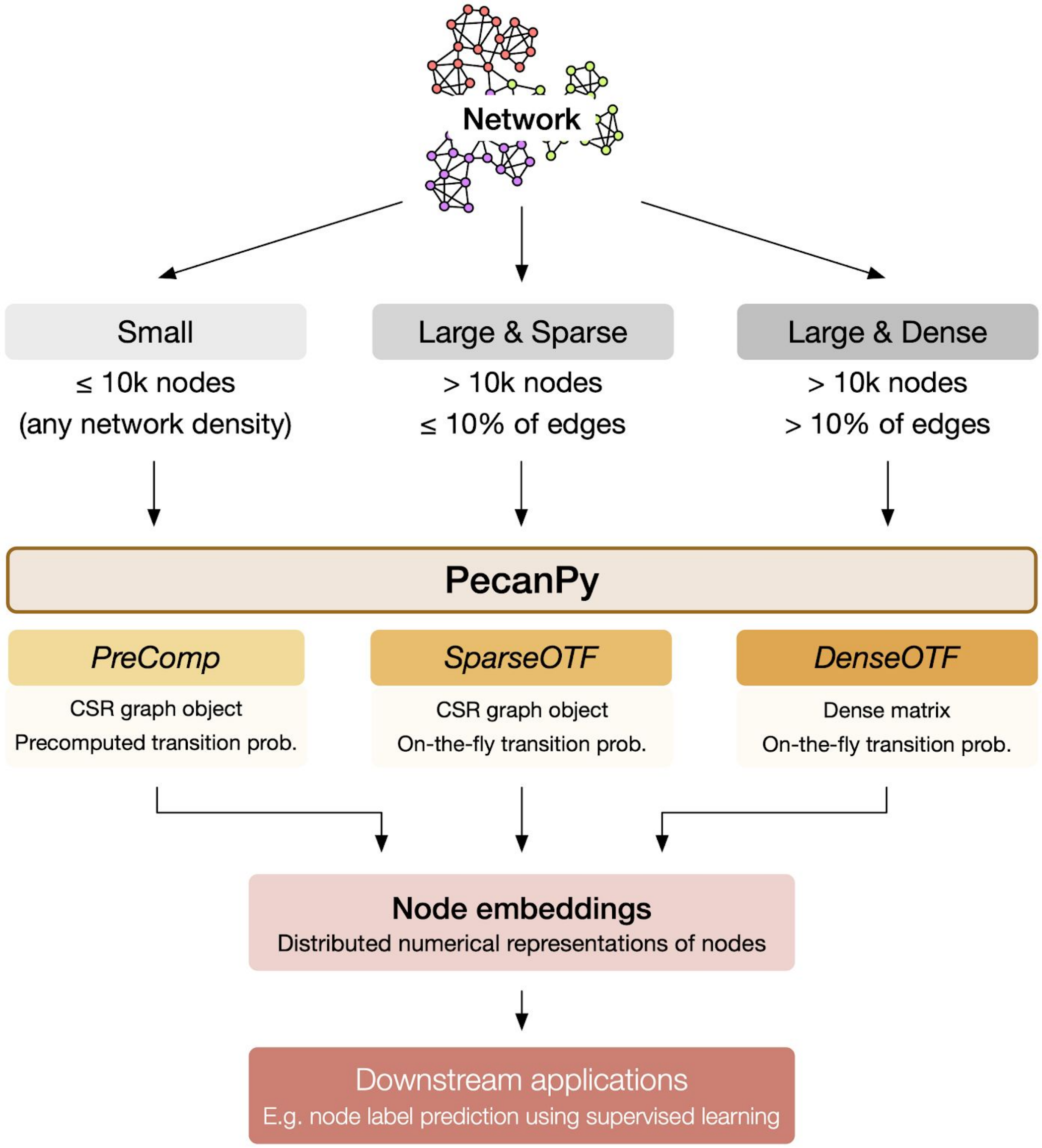

Figure S1. Overview *PecanPy* for fast *node2vec*. PecanPy is a Python implementation of the *node2vec* algorithm. PecanPy can operate in three different modes – *PreComp*, *SparseOTF*, and *DenseOTF* – that are optimized for networks of different sizes and densities; *PreComp* for networks that are small (≤10,000 nodes; any density), *SparseOTF* for networks that are large and sparse (>10,000 nodes and ≤10% of possible edges, i.e. density ≤0.1), and *DenseOTF* for dense networks (>10,000 nodes and >10% of possible edges). These modes appropriately take advantage of compact sparse row (CSR) or dense matrix graph data structures, precomputing transition probabilities (prob.), and computing 2nd-order transition probabilities during walk generation to achieve significant improvements in performance.

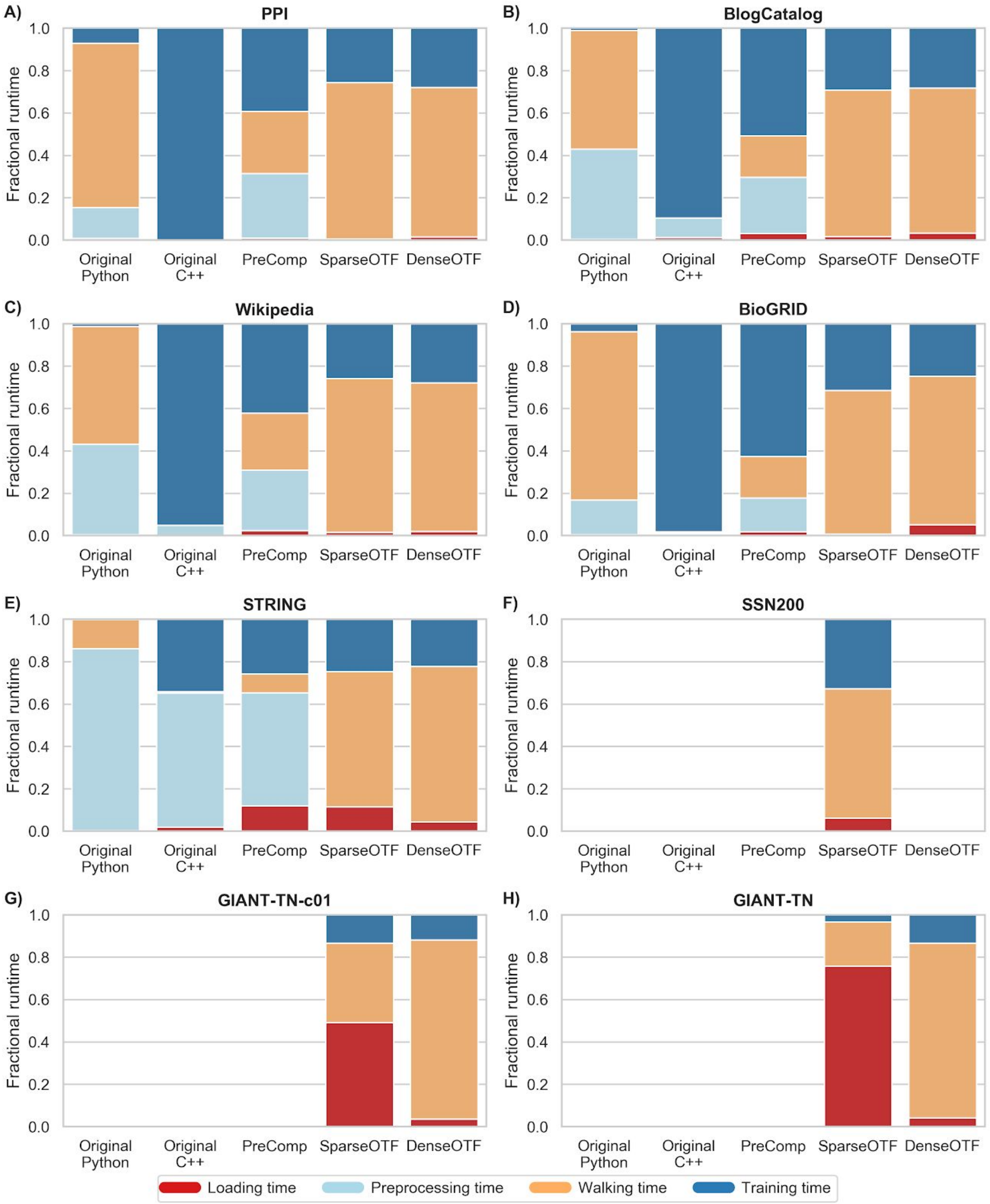

Figure S2. Fraction of runtime contributed by each stage of *node2vec* in different implementations using multiple cores. Each panel corresponds to a single network and each stacked bar within a panel corresponds to an individual *node2vec* implementation. The height of each segment within a bar represents the fraction of runtime contributed by each of the different stages of *node2vec*, tested in a multi-core configuration.

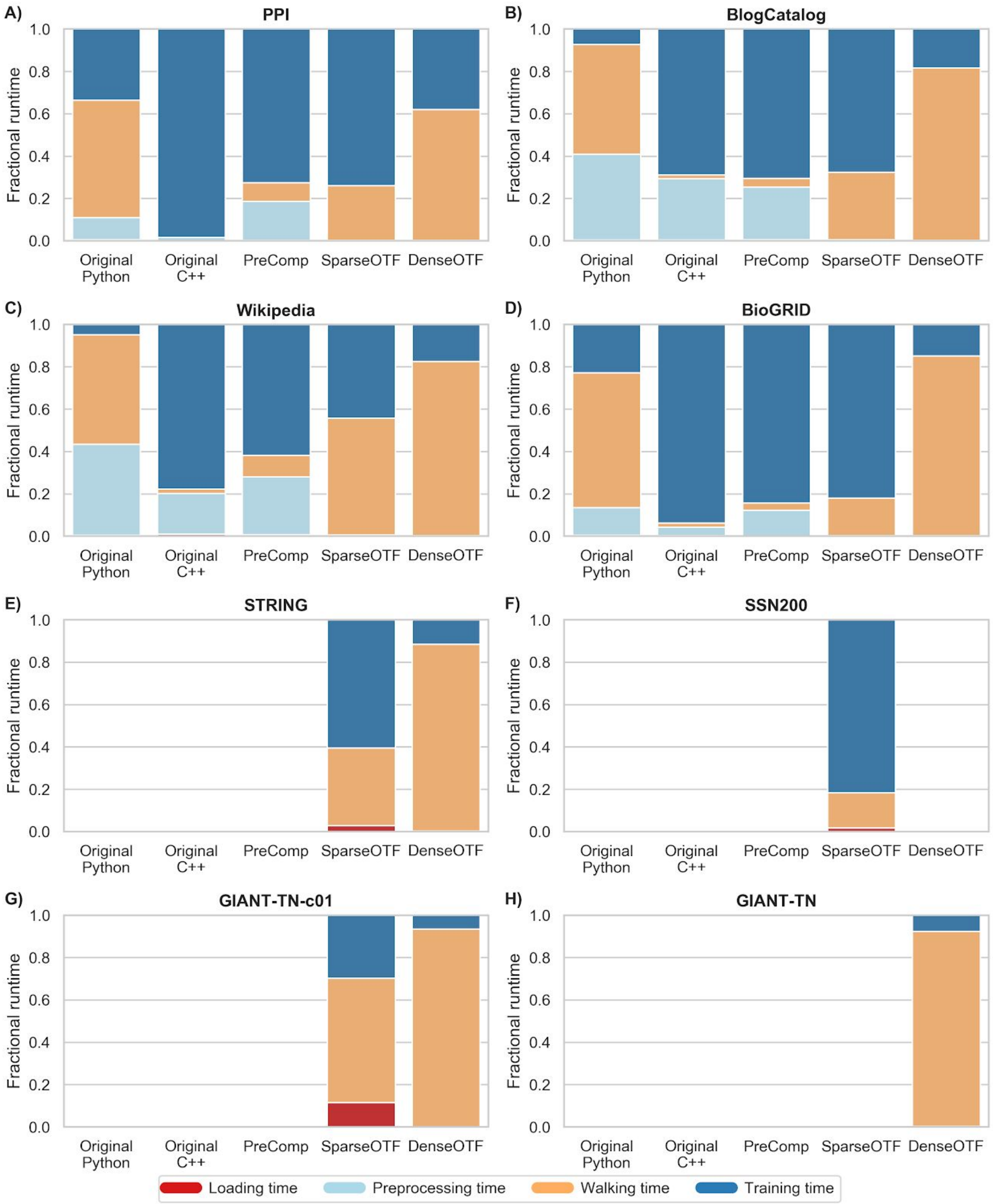

Figure S3. Fraction of runtime contributed by each stage of *node2vec* in different implementations using a single core. Each panel corresponds to a single network and each stacked bar within a panel corresponds to an individual *node2vec* implementation. The height of each segment within a bar represents the fraction of runtime contributed by each of the different stages of *node2vec*, tested in a single-core configuration.

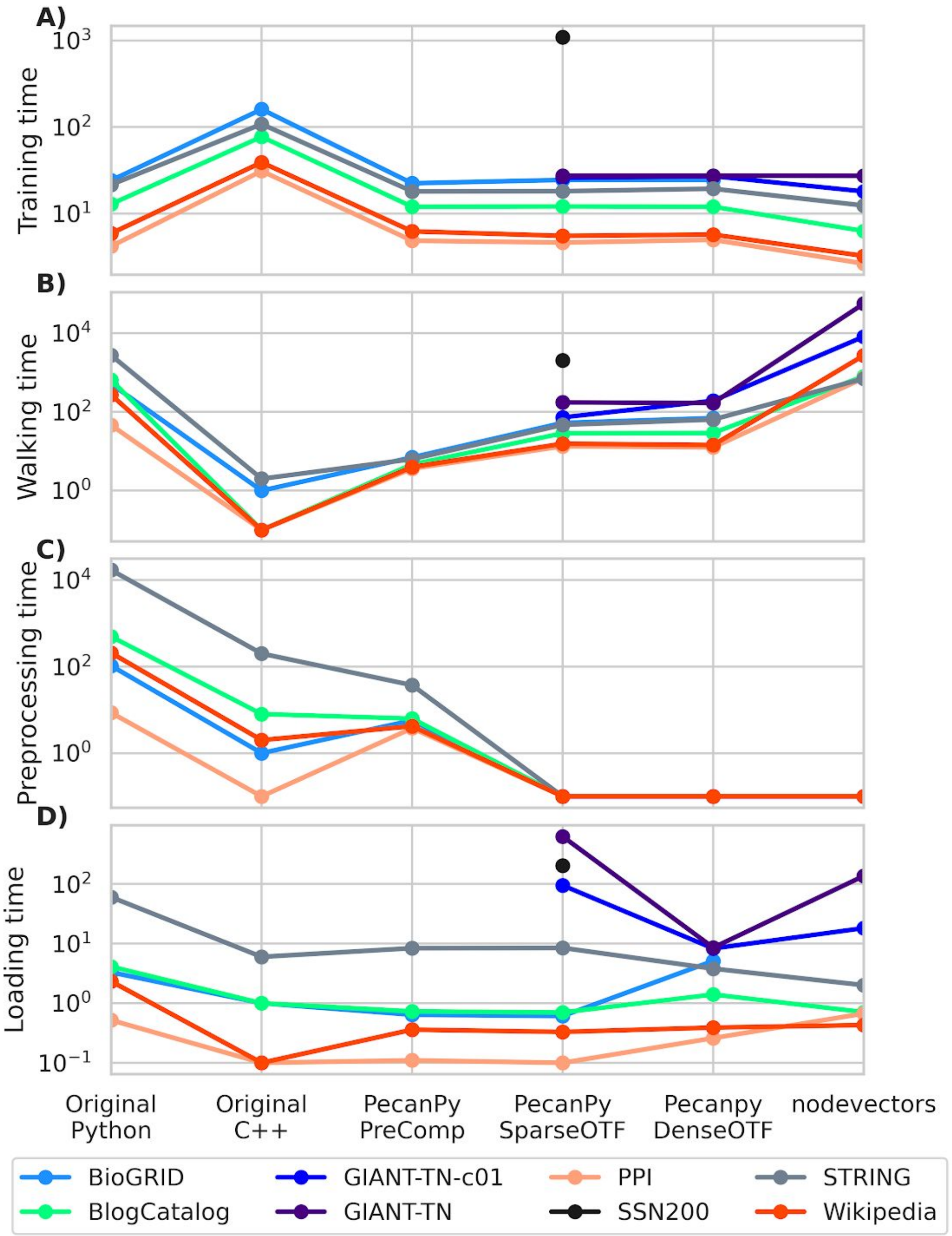

Figure S4. Raw runtimes of each stage of *node2vec* in different implementations using multiple cores. Each parallel plot corresponds to one of four stages of *node2vec*. Each line traces the raw runtime (points on parallel y-axes) of a specific network (color) across the different implementations (x-axis). In all plots, absence of a point for a particular network for any implementation indicates that the network failed to load.

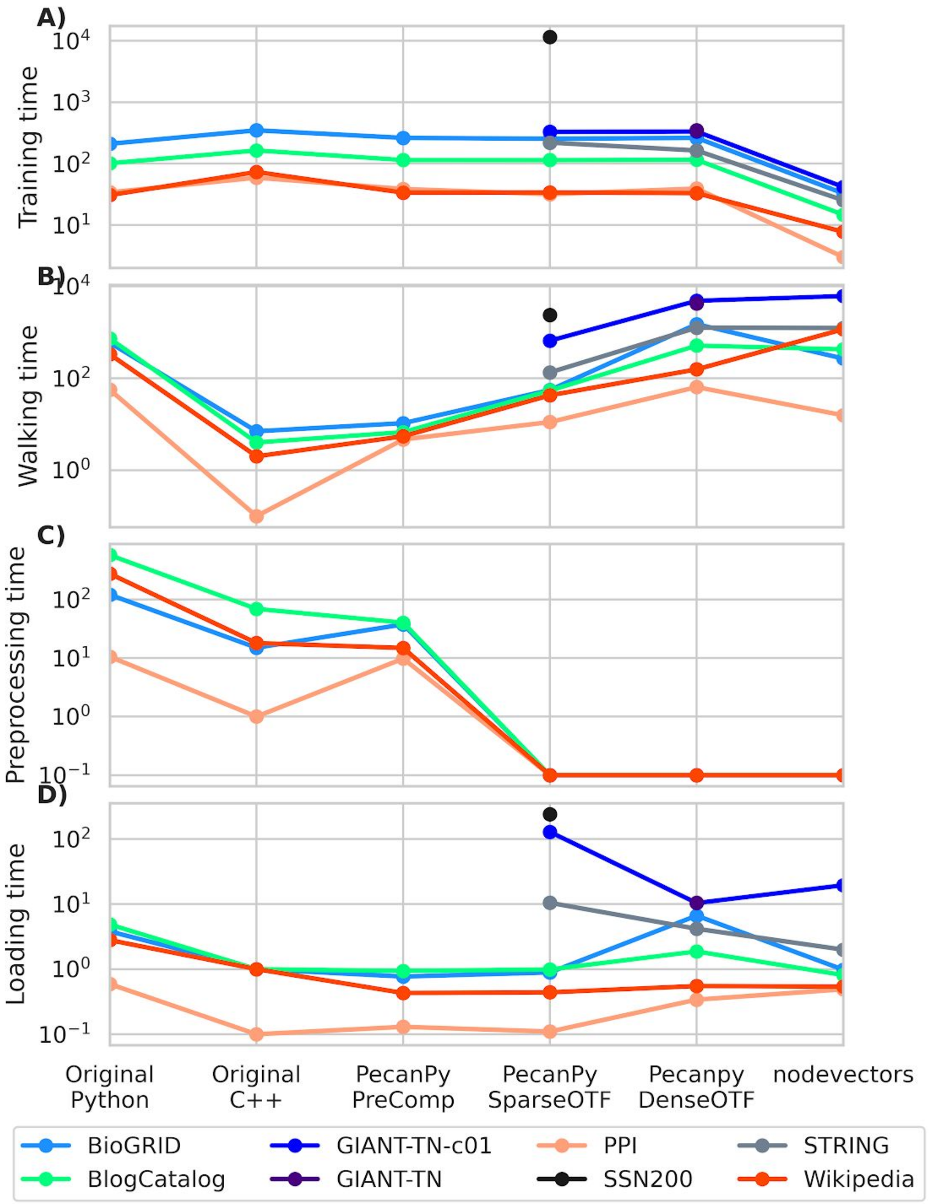

Figure S5. Raw runtimes of each stage of *node2vec* in different implementations using a single core. Each parallel plot corresponds to one of four stages of *node2vec*. Each line traces the raw runtime (points on parallel y-axes) of a specific network (color) across the different implementations (x-axis). In all plots, absence of a point for a particular network for any implementation indicates that the network failed to load.

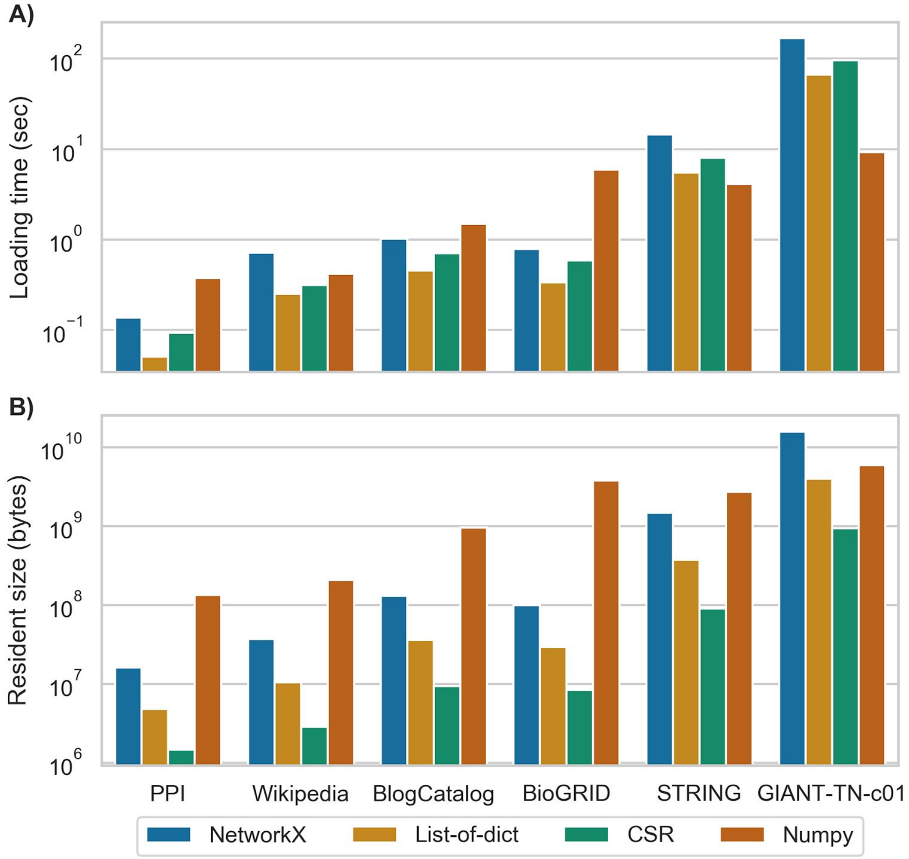

Figure S6. Effect of graph data structure on loading time and memory usage. Plot (A) shows the total time for loading networks, and plot (B) shows the total memory usage. Each plot contains groups of bars, each group corresponding to a network (among seven select networks), and each bar in the group corresponding to a specific network data structure.

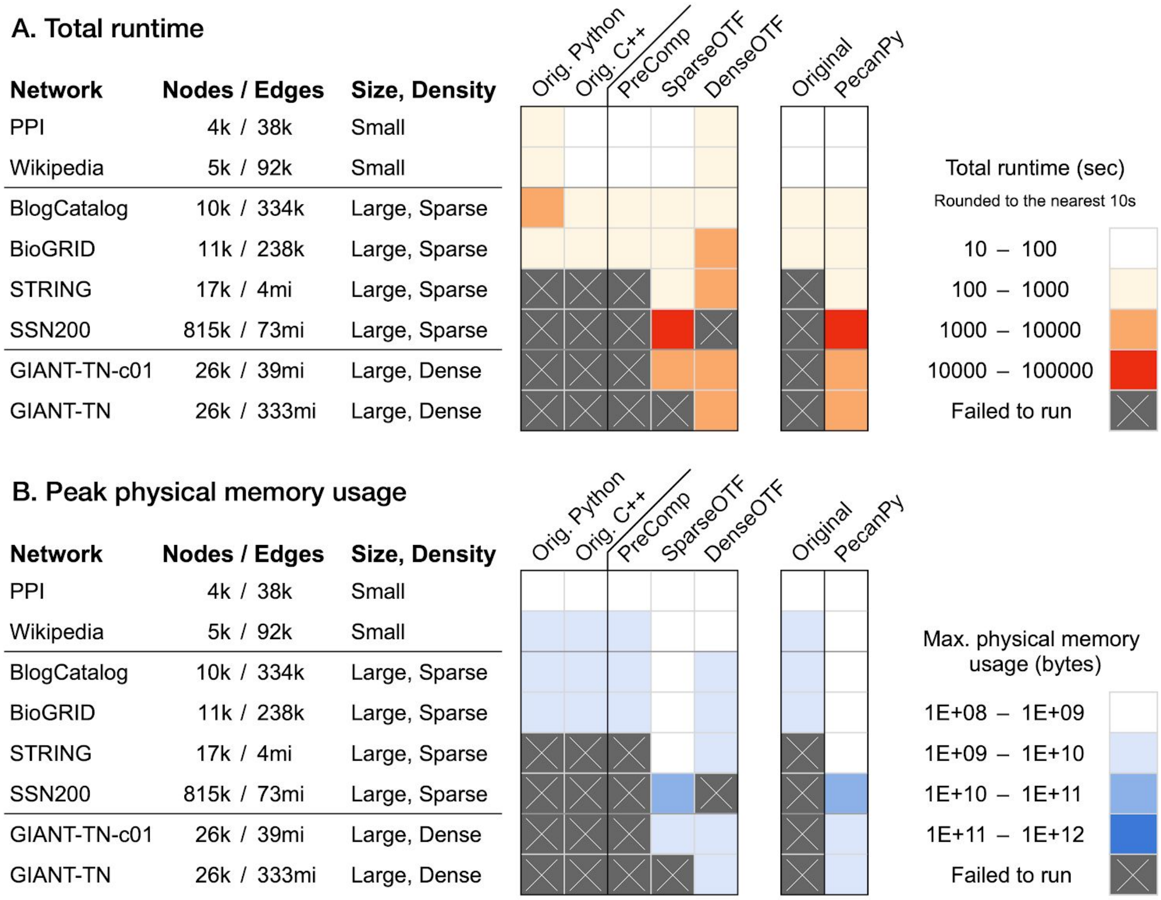

Figure S7. Summary of runtime and memory of PecanPy and the original implementations of *node2vec* using a single core. The eight networks of varying sizes and densities are along the rows. The software implementations are along the columns. The first heatmap (on the left) shows the performance of the original Python and C++ software along with the three modes of PecanPy (*PreComp*, *SparseOTF*, and *DenseOTF*). The adjacent 2-column heatmap (on the right) summarizes the performance of the original (best of Python and C++ versions) and PecanPy (best of *PreComp*, *SparseOTF*, and *DenseOTF*) implementations. Lighter colors correspond to lower runtime in panel A and lower memory usage in panel B. Crossed grey indicates that the particular implementation (column) failed to run for a particular network (row).

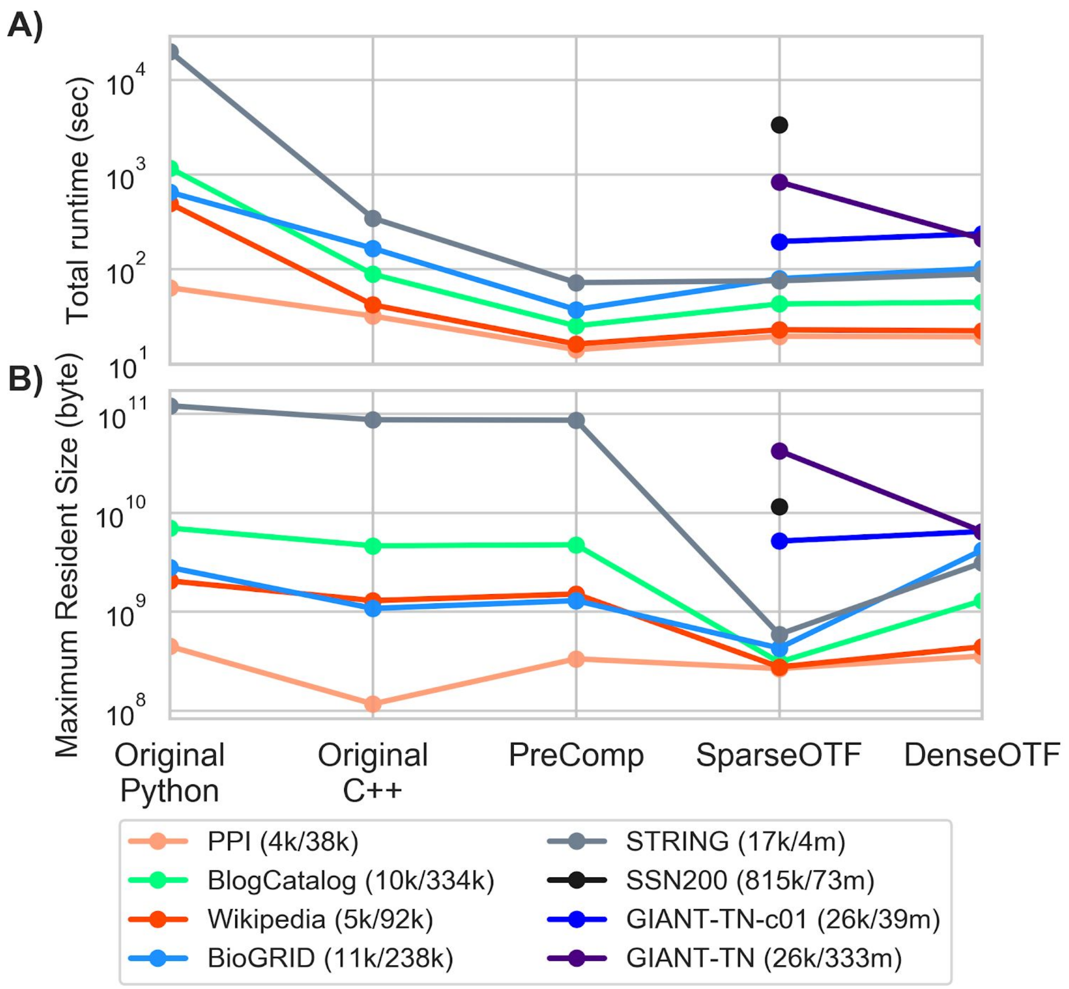

Figure S8. Performance of the original Python and C++ implementations and the three new implementations – *PreComp*, *SparseOTF*, and *DenseOTF* – on eight networks using multiple cores. The parallel plots trace the performance of different *node2vec* implementations (x-axis) for 8 networks (colored dots/lines; number of nodes/edges are in legend below) in terms of (A) total runtime (seconds) and (B) peak memory usage (bytes). Absence of a dot indicates the failure of a particular implementation to run for a particular network.

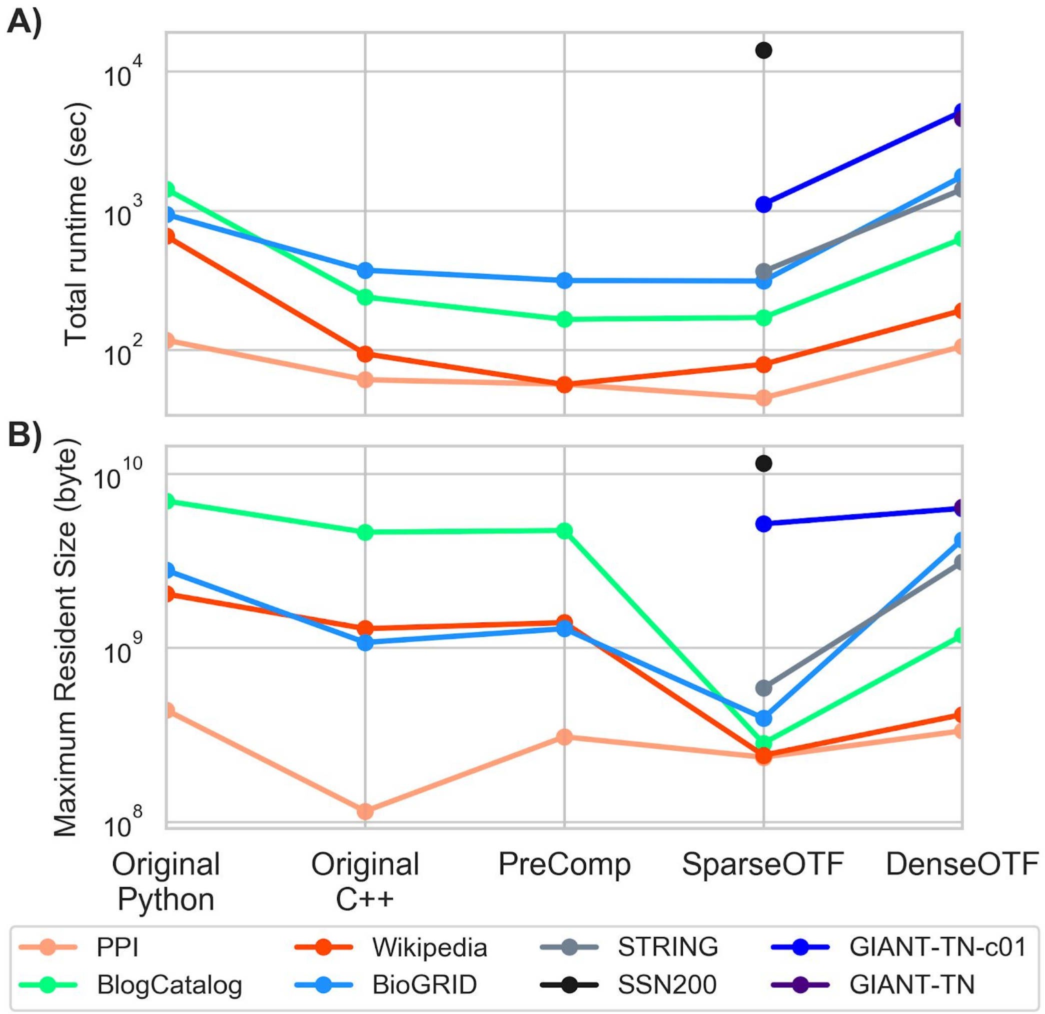

Figure S9. Performance of the original Python and C++ implementations and the three new implementations – *PreComp*, *SparseOTF*, and *DenseOTF* – on eight networks using a single core. The parallel plots trace the performance of different *node2vec* implementations (x-axis) for 8 networks (colored dots/lines) in terms of (A) total runtime (seconds) and (B) peak memory usage (bytes). Absence of a dot indicates the failure of a particular implementation to run for a particular network.

Multi-core

A. Total runtime

Network	Nodes / Edges	Size, Density
PPI	4k / 38k	Small
Wikipedia	5k / 92k	Small
BlogCatalog	10k / 334k	Large, Sparse
BioGRID	11k / 238k	Large, Sparse
STRING	17k / 4mi	Large, Sparse
SSN200	815k / 73mi	Large, Sparse
GIANT-TN-c01	26k / 39mi	Large, Dense
GIANT-TN	26k / 333mi	Large, Dense

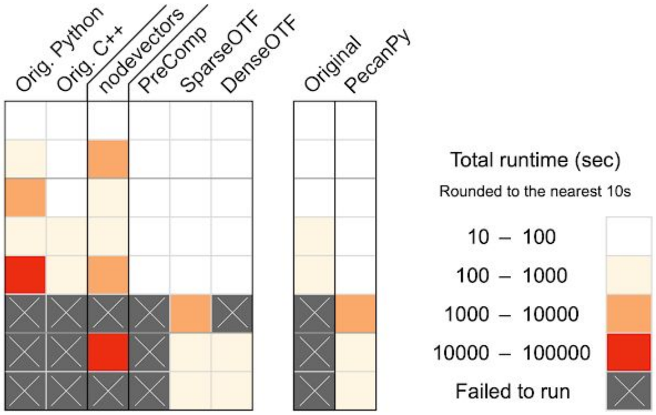

B. Peak physical memory usage

Network	Nodes / Edges	Size, Density
PPI	4k / 38k	Small
Wikipedia	5k / 92k	Small
BlogCatalog	10k / 334k	Large, Sparse
BioGRID	11k / 238k	Large, Sparse
STRING	17k / 4mi	Large, Sparse
SSN200	815k / 73mi	Large, Sparse
GIANT-TN-c01	26k / 39mi	Large, Dense
GIANT-TN	26k / 333mi	Large, Dense

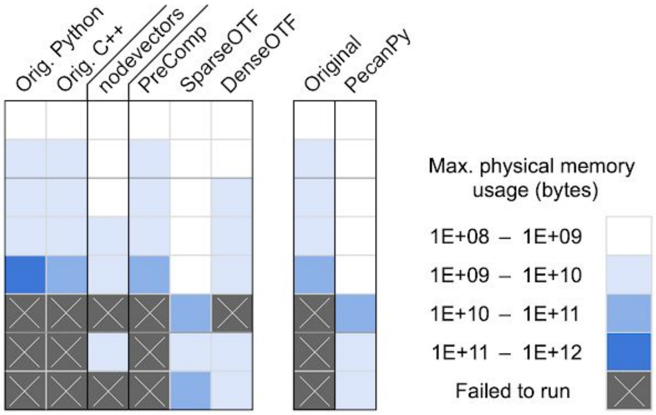

Single-core

C. Total runtime

Network	Nodes / Edges	Size, Density
PPI	4k / 38k	Small
Wikipedia	5k / 92k	Small
BlogCatalog	10k / 334k	Large, Sparse
BioGRID	11k / 238k	Large, Sparse
STRING	17k / 4mi	Large, Sparse
SSN200	815k / 73mi	Large, Sparse
GIANT-TN-c01	26k / 39mi	Large, Dense
GIANT-TN	26k / 333mi	Large, Dense

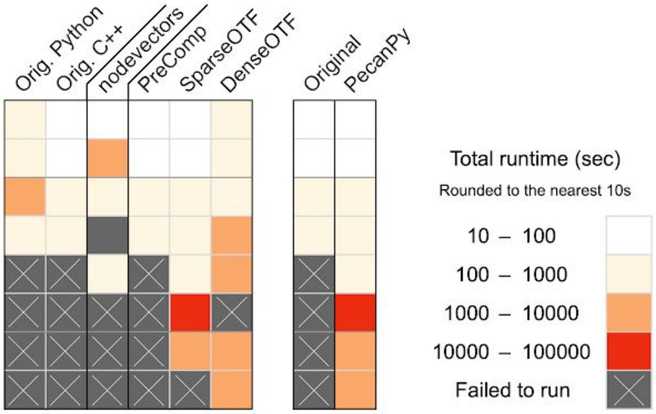

D. Peak physical memory usage

Network	Nodes / Edges	Size, Density
PPI	4k / 38k	Small
Wikipedia	5k / 92k	Small
BlogCatalog	10k / 334k	Large, Sparse
BioGRID	11k / 238k	Large, Sparse
STRING	17k / 4mi	Large, Sparse
SSN200	815k / 73mi	Large, Sparse
GIANT-TN-c01	26k / 39mi	Large, Dense
GIANT-TN	26k / 333mi	Large, Dense

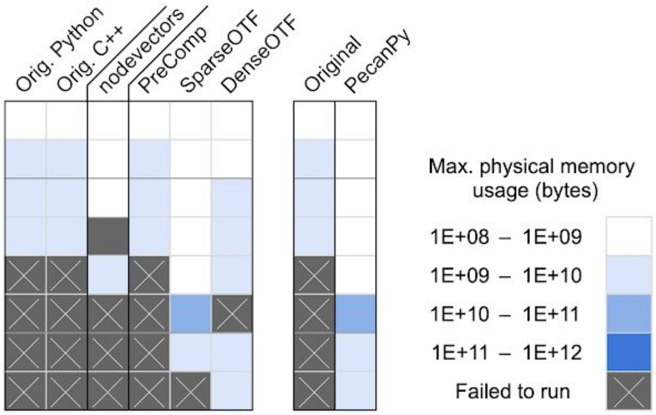

Figure S10. Summary of runtime and memory of PecanPy, nodevectors, and the original implementations of *node2vec* using multiple cores (A and B) and a single core (C and D). The eight networks of varying sizes and densities are along the rows. The software implementations are along the columns. In each panel, the first heatmap (on the left) shows the performance of the original Python and C++ software and the nodevectors software along with the three modes of PecanPy (*PreComp*, *SparseOTF*, and *DenseOTF*). The adjacent 2-column heatmap (on the right) summarizes the performance of the original (best of Python and C++ versions) and PecanPy (best of *PreComp*, *SparseOTF*, and *DenseOTF*) implementations. Lighter colors correspond to lower runtime in panels A and C, and lower memory usage in panels B and D. Crossed grey indicates that the particular implementation (column) failed to run for a particular network (row).

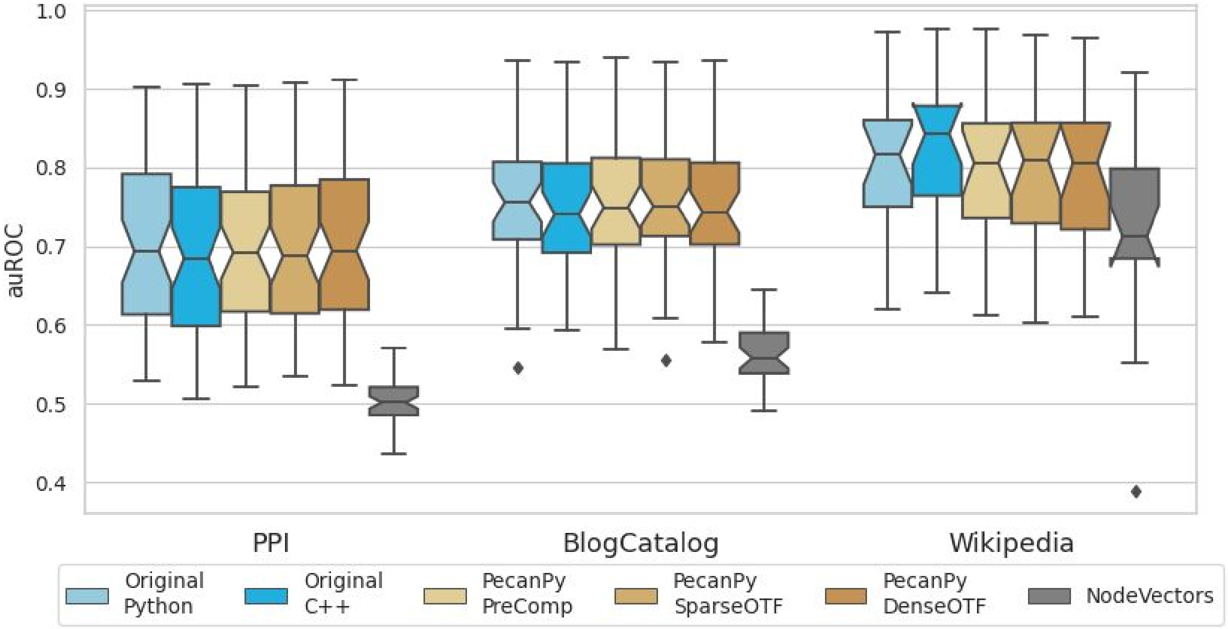

Figure S11. Evaluation of embedding in node classification tasks. Each group of boxplots corresponds to one of three networks. Individual boxplots in a group correspond to the distribution of auROC scores using node embeddings generated using a specific implementation (different colors).

3. Supplementary Tables

Table S1. List of existing implementations of node2vec, all accessed as of February 2021.

Name	Tested	Notes	Link
Original Python implementation	Yes		https://github.com/aditya-grover/node2vec
Original C++ implementation	Yes		https://github.com/snap-stanford/snap/tree/master/examples/node2vec
Nodevectors	Yes	Similar to SparseOTF modes presented above, *nodevectors* computes transition probability on-the-fly, with cache optimized csr graph object. However, it would not be able to achieve good performance for dense networks as discussed above (see Optimization for Dense Networks)	https://github.com/VHRanger/nodevectors
Node2vec with tensor flow	No	Replaced gensim with tensorflow for training embedding from precomputed random walks, which was shown (Zhou et al., 2018) to be the least time consuming step of the algorithm. Thus, we believe this implementation will not be able to produce significant improvement in runtime.	https://github.com/apple2373/node2vec
Python3 implementation of node2vec	No	Same as original Python implementation, but reformatted to be compatible with Python3.	https://github.com/eliorc/node2vec
C++ implementation of node2vec	No	Essentially the original C++ implementation of node2vec with a Python API. It is expected to have the same performance as that of the original C++ implementation.	https://github.com/thibaudmartinez/node2vec
Node2vec implementation in dependency-less C++	No	Same as original C++ implementation of node2vec without other SNAP programs.	https://github.com/xgfs/node2vec-c
Implementation of node2vec with different random distribution sampling method other than alias method	No	Same implementation as the original Python implementation, with numba accelerated random distribution sampling, which does not have any significant impact on runtime performance.	https://github.com/NilsFrahm/Node2vec

Table S2. Summary of *node2vec* implementations

Implementation	Graph data structure	Precompute transition probabilities	Parallelized walk	Non-integer node ID
Original Python	NetworkX	✓	x	x
Original C++	SNAP	✓	✓	x
nodevectors	CSR	x	✓	x
PreComp	CSR	✓	✓	✓
SparseOTF	CSR	x	✓	✓
DenseOTF	Dense Matrix	x	✓	✓

Table S3. Properties of the diverse networks used in this study

Network	Weighted	# Nodes	# Edges	Density	File size
PPI	x	3,852	38,273	5.16E-03	707K
Wikipedia	✓	4,777	92,406	8.10E-03	2.0M
BlogCatalog	x	10,312	333,983	6.28E-03	3.2M
BioGRID	x	20,558	238,474	1.13E-03	2.5M
STRING	✓	17,352	3,640,737	2.42E-02	60M
SSN200	✓	814,731	72,618,574	2.19E-04	2.0G
GIANT-TN-c01	✓	25,689	38,904,929	1.18E-01	1.1G
GIANT-TN	✓	25,825	333,452,400	1.00E+00	7.2G

Table S4. All Runtimes for All Implementations on All Networks. The entries for Total time and Maximum residence size that are colored dark grey indicate cases when the implementation in the row failed, at which point these data (time and size) were recorded.

Table S5. Wilcoxon statistics for embedding evaluation. Wilcoxon paired test is performed on the auROC score obtained by using the embedding generated using each implementation against that from the original Python implementation.

(Table S4. and Table S5. are provided as tsv files)

4. Supplementary References

Finlayson,S.G. et al. (2014) Building the graph of medicine from millions of clinical narratives. Sci. Data, 1, 140032.

Greene,C.S. et al. (2015) Understanding multicellular function and disease with human tissue-specific networks. Nat. Genet., 47, 569–576.

Grover,A. and Leskovec,J. (2016) node2vec: Scalable Feature Learning for Networks. ArXiv160700653 Cs Stat.

Hagberg,A. et al. (2008) Exploring network structure, dynamics, and function using networkx Los Alamos National Lab. (LANL), Los Alamos, NM (United States).

Hamilton,W.L. et al. (2018) Representation Learning on Graphs: Methods and Applications. ArXiv170905584 Cs.

Law,J.N. et al. (2019) Accurate and Efficient Gene Function Prediction using a Multi-Bacterial Network. bioRxiv, 646687.

Leskovec,J. and Krevl,A. (2014) SNAP Datasets: Stanford Large Network Dataset Collection.

Liu,R. et al. (2020) Supervised-learning is an accurate method for network-based gene classification. Bioinformatics.

Mikolov,T. et al. (2013) Distributed Representations of Words and Phrases and their Compositionality. ArXiv13104546 Cs Stat.

Nelson,W. et al. (2019) To Embed or Not: Network Embedding as a Paradigm in Computational Biology. Front. Genet., 10.

Perozzi,B. et al. (2014) DeepWalk: Online Learning of Social Representations. Proc. 20th ACM SIGKDD Int. Conf. Knowl. Discov. Data Min. - KDD 14, 701–710.

Stark,C. et al. (2006) BioGRID: a general repository for interaction datasets. Nucleic Acids Res., 34, D535–D539.

Szklarczyk,D. et al. (2015) STRING v10: protein–protein interaction networks, integrated over the tree of life. Nucleic Acids Res., 43, D447–D452.

Walt,S. van der et al. (2011) The NumPy Array: A Structure for Efficient Numerical Computation. Comput. Sci. Eng., 13, 22–30.

Yue,X. et al. (2020) Graph embedding on biomedical networks: methods, applications and evaluations. Bioinformatics, 36, 1241–1251.
